# Efficient data reduction for time-of-flight neutron scattering experiments on single crystals

**DOI:** 10.1107/S1600576722009645

**Published:** 2022-11-04

**Authors:** Andrei T. Savici, Martyn A. Gigg, Owen Arnold, Roman Tolchenov, Ross E. Whitfield, Steven E. Hahn, Wenduo Zhou, Igor A. Zaliznyak

**Affiliations:** a Neutron Scattering Division, Oak Ridge National Laboratory, Oak Ridge, TN, USA; b ISIS Facility, Rutherford Appleton Laboratory, Chilton, Didcot, UK; c Tessella Ltd, Abingdon, UK; d Computer Science and Mathematics Division, Oak Ridge National Laboratory, Oak Ridge, TN, USA; e Condensed Matter Physics and Materials Science Division, Brookhaven National Laboratory, Upton, NY, 11973, USA; Lund University, Sweden; Keele University, United Kingdom

**Keywords:** time-of-flight neutron scattering, algorithms, single crystals

## Abstract

Event-mode data collection presents remarkable new opportunities for time-of-flight neutron scattering studies of collective excitations, diffuse scattering from short-range atomic and magnetic structures, and neutron crystallography. In these experiments, large volumes of the reciprocal space are surveyed, often using different wavelengths and counting times. These data then have to be added together, with accurate propagation of the counting errors. This paper presents a statistically correct way of adding and histogramming the data for single-crystal time-of-flight neutron scattering measurements. In order to gain a broader community acceptance, particular attention is given to improving the efficiency of calculations.

## Abstract 2

In neutron scattering experiments, data sets with different statistical significance are collected. A new method is presented to efficiently calculate the weights of single-crystal time-of-flight measurements, and to add together the various contributions.

## 1. Introduction

The advent of time-of-flight (TOF) neutron scattering technology using instruments with large-area pixelated detectors (Perring *et al.*, 1994 ▸; Ewings *et al.*, 2019 ▸; Bewley *et al.*, 2006 ▸; Stone *et al.*, 2014 ▸; Ye *et al.*, 2018 ▸; Jogl *et al.*, 2011 ▸; Nakajima *et al.*, 2011 ▸; Kajimoto *et al.*, 2011 ▸) has revolutionized the field of neutron scattering experimentation. At the same time, it has introduced profound challenges for data processing. The very essence of the TOF method poses an existential problem for the traditional paradigm of measuring intensity in a neutron experiment. Namely, assigning neutron intensity simultaneously to a position on a detector and a finite time interval can be all but impossible because a neutron’s trajectory in time can pass across several detector pixels if the time interval is long enough (Perring, 1999 ▸). This gives rise to the problem of partitioning the time-histogrammed neutron count among detector pixels. In the past, this problem was unavoidable because detected neutrons were time-binned in hardware due to the lack of computing resources and the resulting intensities were stored as spectral histogram files (SPE file format).

Since the result of the data processing is a histogram in sample reciprocal space, any partial histogramming that occurs before that final histogram is obtained, and which requires further histogramming in order to obtain it, will be referred to as pre-histogramming. Adding intensities of time-histogrammed spectra while accurately accounting for statistical errors is quite problematic. Once the data are pre-histogrammed, the intensities need to be properly normalized; one also needs to account for detector efficiency and the solid angle covered by detector pixels to which the time bin is assigned, and such assignment is often ambiguous. The problem of adding the pre-histogrammed data while precisely propagating the errors is aggravated for weak, diffuse and inelastic signals where the intensity in each time bin is low, often 1 or 0, and statistical uncertainty cannot be reliably evaluated. Consequently, various approximations and specific workflows were developed for adding and histogramming the pre-histogrammed data that are still used in present-day software packages (Azuah *et al.*, 2009 ▸; Ewings *et al.*, 2016 ▸; Inamura *et al.*, 2013 ▸).

In processing the TOF spectroscopy data, an approximation has often been made that all histogram bins within a given region of reciprocal space have equal statistical significance. Then, these bins are simply averaged as if they represent independent identical measurements of the differential scattering cross section. This is a coarse approximation, since detectors have different efficiencies and bin sizes are uneven (the same ranges in TOF are not the same ranges in energy transfer, since the velocity is also a function of detector position), and especially when measurements are made with unequal flux or measurement time: the statistical noise of the average data set is dominated by the data with the worst statistics. This is a known deficiency of some widely used software packages which average the pre-histogrammed data.

**Figure 1:**
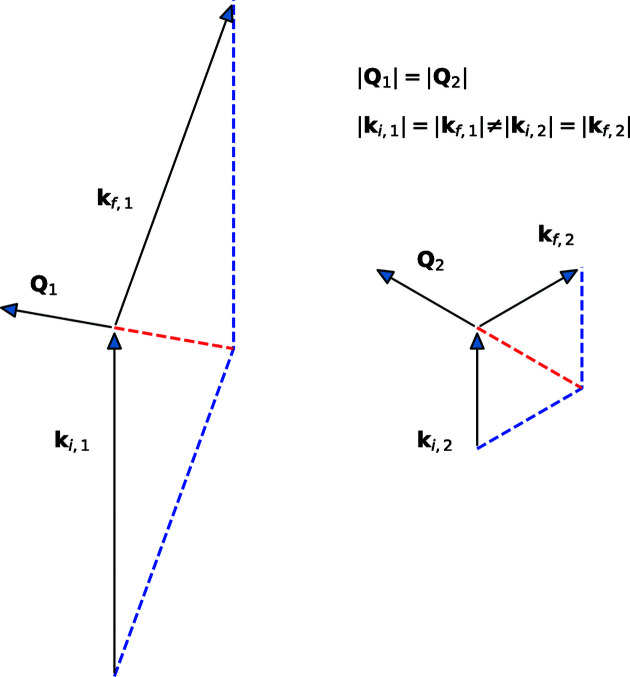
In diffraction experiments, one can measure the same magnitude momentum transfer 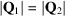 in different detectors. From the conservation of momentum in the two cases one can find that different wavelengths (or wavevectors) were used. In addition, the orientation of the two momentum transfer vectors is different, so in order to satisfy the Bragg diffraction conditions the sample must be rotated. The incident flux is generally different at different wavelengths.

In the early days of TOF diffraction, Bragg peak intensities were obtained by integrating neutron count within a narrow time window and a small detector area (Windsor, 1981 ▸). A normalization factor, which accounts for the detector solid angle and the wavelength-dependent flux, was then applied to the integrated peak intensity in order to obtain the differential scattering cross section. The assumption made in this case is that the entire peak is measured with a single neutron wavelength (no spread). In modern TOF diffractometers, however, one can measure the same peak with different sample orientations, at different wavelengths, as shown in Fig. 1 ▸. In general, the incident flux varies as a function of wavelength. As a consequence, the intensity of the same peak measured at different wavelengths and sample orientations must be considered separately. In the case of short-range diffuse scattering, the features of interest are sometimes orders of magnitude broader than a Bragg peak (1–10 Å^−1^ versus 0.1 Å^−1^). Clearly, such an approach would not work well, since measurements of large volumes of reciprocal space combine data from different neutron wavelengths.

In the modern incarnation of the TOF technology implemented at the Spallation Neutron Source (SNS), the event mode of data collection is used (Granroth *et al.*, 2018 ▸). Remarkably, the event mode parts with the traditional concept of measuring intensities in a neutron experiment. Instead, information about each detected neutron is stored in the form of a descriptive entry in a database, where each neutron is tagged with its arrival time at the detector. For event mode to be meaningful, the detector time resolution should be such that the spatial extent of the neutron’s trajectory within the time resolution window is smaller than the detector pixel. For a thermal neutron traveling across a 1′′ detector, with a speed of 2500 m s^−1^, event mode requires time resolution δ*t* < 10 µs. At the SNS, the neutron events are time-stamped to a precision of 0.1 µs, well within this requirement (Berry, 2016 ▸).

The aim of this paper is to present an efficient algorithm for the TOF neutron data reduction, which focuses on statistically rigorous normalization, combination and histogramming of the event data. This approach clearly separates the workflow into two independent tasks. The first is that of transforming data coordinates, and it is carried out entirely on the event data (Peterson *et al.*, 2015 ▸, 2018 ▸). The second task is that of creating a histogram by binning the event data, and is carried out simultaneously with weighting the bins by a normalization factor depending on the measurement statistics.

The central task addressed by the present algorithm is that of computing the statistical weights for the measured events, with the highest possible mathematical rigor. This task is at the core of comparing and combining different event data sets. These weights are similar to the monitor count that is used for normalization and adding different measurements in traditional triple-axis neutron spectroscopy (Shirane *et al.*, 2002 ▸). When the data are combined, or coarse-grained, the neutron counts are added and the corresponding monitors are separately combined for the normalization. Similarly, in our approach, when events are histogrammed the total statistical weight is evaluated for each bin and then used for the normalization of the total count in the bin. At the SNS, the present algorithm is already in use for diffuse elastic scattering data from single crystals and is gaining traction in the inelastic community. We present particular examples of single-crystal diffuse scattering (both elastic and inelastic) as a motivation for the new approach, as well as details of its practical implementation.

The paper is organized as follows. Section 2 presents a summary of theoretical aspects of neutron measurement and the UB-matrix formalism. An overview of the existing approaches to single-crystal TOF data reduction is given in Section 3. Section 4 describes our new algorithm for efficient weighted normalization and histogramming of the event TOF data, and Section 5 presents its practical implementation and examples. In conclusion, we briefly summarize the main features and advantages of our approach.

## 2. Theoretical aspects of single-crystal neutron scattering measurements

By selecting a particular neutron energy transfer, 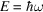 , a neutron spectrometer measures the double differential scattering cross section, 

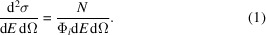

Here, *N* is the number of neutrons measured in a certain time interval, in a detector covering a solid angle of 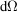 , within an energy transfer range d*E*, and 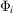 is the corresponding time-integrated neutron flux of the incident beam during the measurement. According to Van Hove (1954 ▸), the scattering cross section is proportional to the dynamical structure factor, 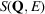 , 

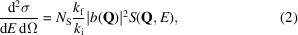

where 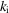 and 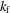 are the magnitudes of the incident and scattered neutron wavevectors, respectively, 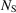 is the number of unit cells in the sample, and 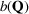 is their scattering length (Squires, 2012 ▸; Zaliznyak & Lee, 2005 ▸). If one is interested only in the single time (instantaneous) correlations of magnetic moments or atomic positions, the energy transfer in equation (1) can be integrated out to obtain the (single) differential cross section, 

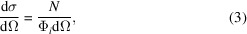

which is proportional to the static structure factor, 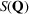 .

### 2.1. Kinematics of the scattering process

From the conservation of momentum and energy, the momentum and energy transfer to the sample are

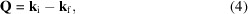




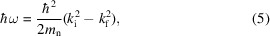

where 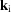 and 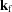 are the incident and scattered neutron wavevectors, respectively, and 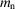 is the neutron mass. The magnitudes of the wavevectors, 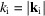 and 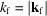 , are proportional to the corresponding neutron speeds:

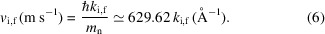

The momentum transfer, 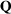 , is determined by the incident and final neutron velocities, and the detector position. For spectrometers, where either the incident or final neutron energy is fixed, the variable velocity is usually replaced by the energy transfer. For TOF instruments at a pulsed source, the neutron energy is thus determined using the TOF with respect to a neutron start time from the source, which is defined by the source pulse.

Diffractometers use the time interval between neutron production and neutron detection to calculate the neutron velocity, knowing the path from the source to detector, via the sample. The assumption is that the scattering is fully elastic, so the velocity is constant along the neutron flight path.

In direct-geometry spectrometers, the incident energy can be selected using choppers, devices that allow neutrons to pass through only at certain time intervals (Windsor, 1981 ▸). Knowing the instrument geometry, from the time difference between the moment neutrons leave the source and the moment they pass through the chopper, one can find the initial neutron velocity and therefore the time when neutrons arrive at the sample position. Since the total TOF is recorded for each neutron, one can calculate the final velocity from the time interval between the time a neutron is scattered by the sample and the time it is detected. A similar reasoning applies for indirect-geometry spectrometers, where the final energy is fixed instead.

### 2.2. Coordinate transformation of the event data and UB-matrix formalism

For each detected neutron, the information about the incident and the scattered states is recorded in the event data file. The incident and the scattered neutron wavevectors are calculated in the laboratory reference frame of the instrument, and used in equations (4) and (5). However, for neutron scattering from a crystal, the relevant reference frame for the momentum transfer, 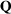 , is the one co-aligned with the reciprocal-lattice vectors of the crystal. Hence, an important primary task of the data reduction is to perform a coordinate transformation that translates neutron events from the instrument laboratory frame to a coordinate frame of the crystal.

Due to the conservation of energy and momentum, the detector trajectories in the reciprocal space of the crystal cover only a limited part of this space [see *e.g.* Ewings *et al.* (2016 ▸)]. For a stationary crystal, detectors of a spectrometer subtend a 3D surface in the 4D 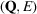 space. To cover the 4D regions of interest, single-crystal neutron scattering instruments rotate the sample with respect to the laboratory frame using goniometers. This introduces a sample-angle-dependent coordinate transformation from the laboratory to the sample reference frame for the events that comprise a single rotational data set.

The physics in the reciprocal space of the crystal can usually be described by a triplet of indices, 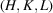 , which measure the wavevector transferred to the crystal in units of its reciprocal lattice. Following the UB-matrix formalism (Busing & Levy, 1967 ▸; Lumsden *et al.*, 2005 ▸), the connection between the momentum transfer in the crystal and the laboratory frame is given by

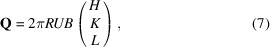

where *R*, *U* and *B* are 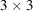 matrices. *B* transforms the reciprocal-lattice coordinates into an orthogonal coordinate system with the first axis along 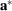 , the second axis perpendicular to 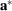 in the 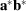 plane and the third axis perpendicular to this plane. This transformation also re-scales the axes to units of Å^−1^. *U* is an orientation matrix describing how the crystal is mounted on the goniometer, and thus transforms the wavevectors to the goniometer coordinate frame at their nominal position. The rotation matrix *R* describes rotations of the crystal reciprocal space around the goniometer axes.

**Figure 2:**
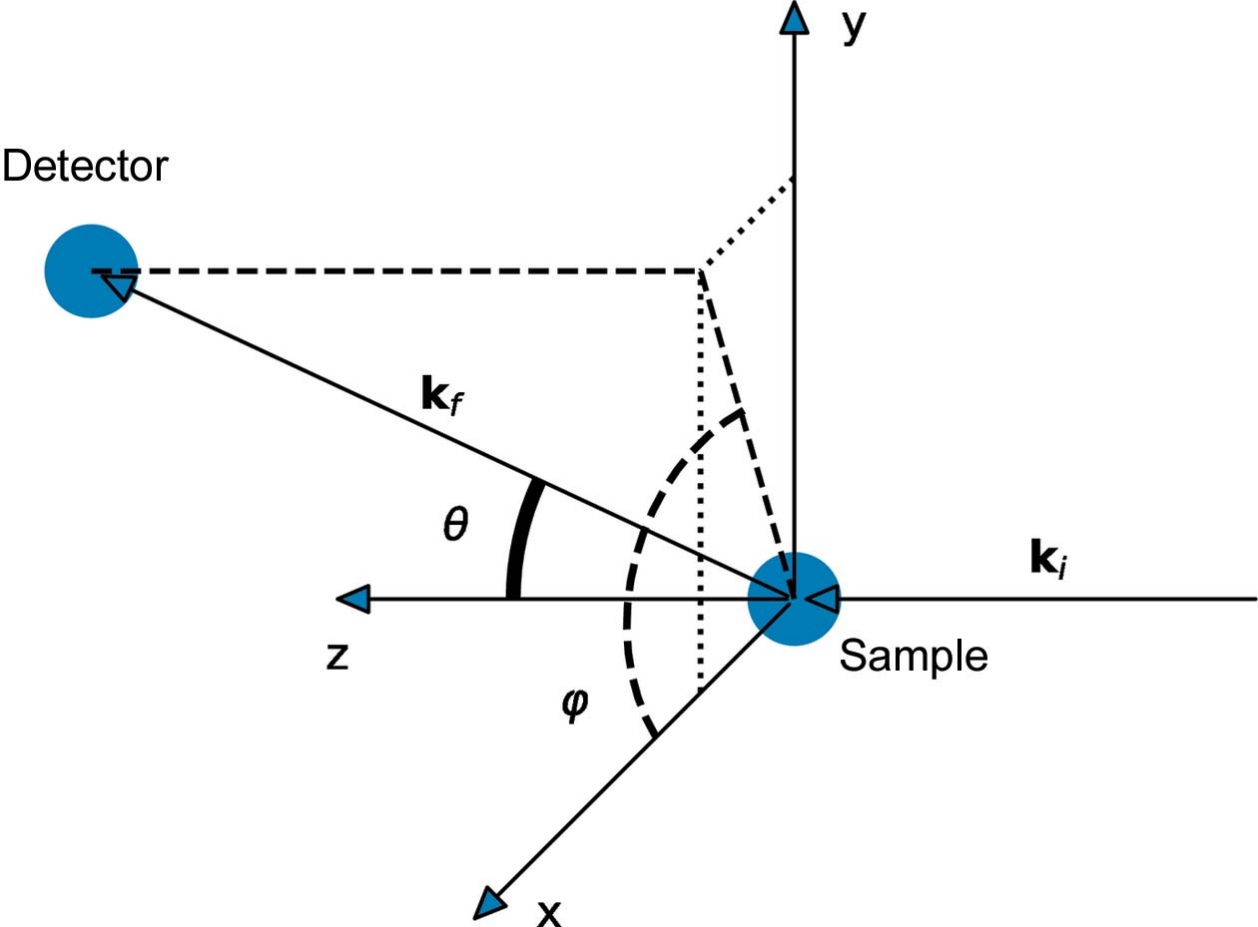
The coordinate system has the incident beam along the *z* axis. The vertical direction is along *y*. The detector position is described in spherical coordinates by polar angle θ, with respect to the *z* axis, and the azimuthal angle φ, the angle between the projection of the detector position in the *xy* plane and the *x* axis.

In the rest of this paper, we will use a right-handed laboratory reference frame with the 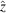 axis along the incident beam, the 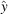 axis vertical up and the 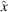 axis in the horizontal plane, perpendicular to 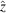 and 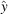 , consistent with the convention adopted at the SNS. The detector position is given by the polar angle θ and the azimuthal angle φ, as shown in Fig. 2 ▸. We can then rewrite equations (4) and (7) as

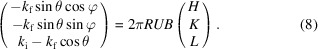

Note that most crystallography papers use the momentum transfer for the neutron (not the sample) (Giacovazzo, 1992 ▸). Equation (4) would be rewritten as 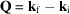 , so there would be a sign inversion of 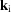 and 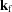 in equation (8).

While this transformation is nominally straightforward, the transformation of the statistical weights of neutron events should be performed carefully. Statistical weights (‘monitors’) account, among other things, for the volume element occupied by the detector pixel(s) where the neutron was detected, so these weights need to be corrected for the Jacobian of the coordinate transformation. This Jacobian is a non-trivial object and its efficient calculation is a task accomplished by the algorithm presented here.

## 3. Data reduction and histogramming of TOF measurements on single crystals

Historically, measurements of the differential neutron scattering cross sections in crystals were performed using single-detector instruments, such as a four-circle diffractometer or a triple-axis spectrometer, with a single detector that moves to the desired positions in the sample reciprocal space. In such a measurement, the data are collected at well defined points on a regular grid and with the statistical accuracy determined by the incident neutron current measured by the monitor. When a number of different measurements are combined, the total neutron count is normalized by the total monitor count, thus improving the data statistical accuracy. This is equivalent to combining the statistical weights of the added intensities.

The introduction of TOF instruments with large detector arrays for inelastic (MARI, MAPS, LET, ARCS, SEQUOIA, CNCS, AMATERAS, 4SEASONS) and diffuse elastic scattering (SXD, WISH, TOPAZ, CORELLI) measurements (Perring *et al.*, 1994 ▸; Ewings *et al.*, 2019 ▸; Bewley *et al.*, 2006 ▸; Stone *et al.*, 2014 ▸; Keen *et al.*, 2006 ▸) presented a new challenge for data reduction and visualization. Different detectors cover parts of the reciprocal space in a nonlinear and often overlapping way. For TOF diffractometers, there is an additional complication in that the flux changes as a function of neutron wavelength. Hence, interpolating the measurements onto a regular grid by adding the neutron counts and combining the corresponding statistical weights presents a challenge. The problem is aggravated in the case of time-histogrammed spectra, where accurately evaluating the statistical weights of the histogrammed intensity is difficult, or impossible (*e.g.* for bins with zero intensity).

### 3.1. Equal-weight approximation for time-histogrammed data

**Figure 3:**
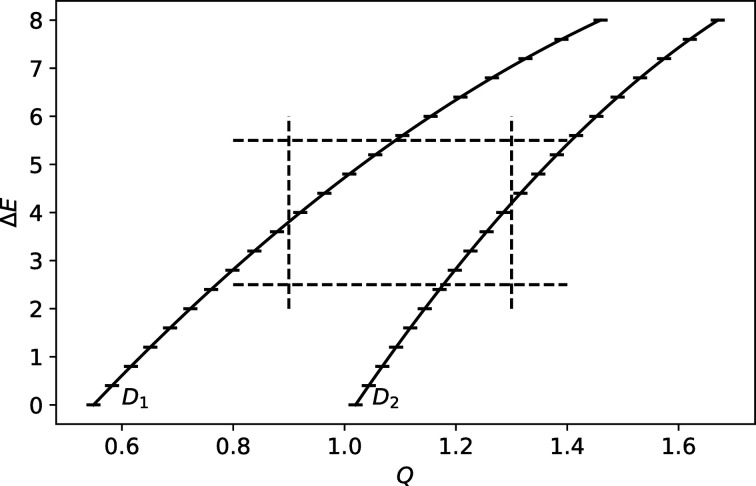
Schematic representation of the histogrammed spectrum approach for inelastic measurement. Energy and momentum transfer are in arbitrary units. Some arbitrary detector trajectories ( 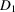 and 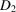 ) are shown. Each energy transfer histogram bin (bin boundaries are marked by the horizontal ticks along the trajectories), in every detector, is considered as an independent measurement of the differential scattering cross section. The intensity in each voxel in the reciprocal space (bounded by dashed lines) is then an average of contributions from the enclosed values.

One simple approach used for processing the pre-histogrammed TOF data is to assume equal weights for all intensities in a histogram bin. This approach is implemented in *MSlice* (Coldea, 2000 ▸) and widely used derived programs, such as *DAVE MSlice* (Azuah *et al.*, 2009 ▸) and *Horace* (Ewings *et al.*, 2016 ▸). The time-histogrammed spectra are transformed into a set of histograms in energy transfer ( 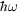 ), one for each detector. When normalized by the solid angle/detector efficiency, and by the incident flux, the intensity in each histogram bin represents an individual measurement of the scattering cross section. For every spectrum in each histogram bin, the momentum transfer is calculated from the corresponding detector position and the energy transfer. The calculated intensities are then assigned to hyper-rectangles on a regular grid in the 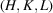 and 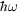 space (voxels). Finally, all contributions in each individual voxel are averaged. A schematic representation is shown in Fig. 3 ▸. The intensity in a particular voxel corresponds to a statistical average with equal weights, 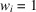 , 

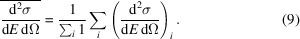

Here, the summation is over all histogram bins for all sample orientations that contribute to the voxel. A similar formula for diffraction experiments, used for example by Welberry *et al.* (2005 ▸), is written as

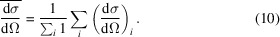




The main deficiency of this algorithm is that it assumes equal weights, *i.e.* that, after efficiency calibration and flux normalization, all measurements are equally relevant statistically. This is very often not the case. First, the detector efficiency varies across the detector array and also in a way that depends on neutron final energy; some detectors may be partially obscured by other elements of the instrument setup and thus have lower effective efficiency, resulting in a larger measurement error. Second, the amount of time (neutron current) over which each sample configuration is measured might generally be different. Partitioning the time-histogram bin between different detectors also presents a challenge. If one averages a measurement with small error bars with one having large errors, the result is noisier and with larger uncertainties than the best measurement. At the minimum, this places a restrictive requirement in the pre-histogrammed scan that all angles must be counted equally. In the worst case, in TOF diffraction, for the different wavelengths contributing to a voxel the neutron flux can differ by an order of magnitude, leading to a large disparity in the error bars (Michels-Clark *et al.*, 2016 ▸). A similar example for direct-geometry spectroscopy measurements will be shown in Section 5.4. In the past, this problem was ‘solved’ by discarding the measurements with larger errors, even though they might contain useful and perhaps unique information. Clearly, such an approach presents a marked handicap for an efficient data analysis.

There are also significant penalties to pay for pre-histogramming the data from the point of view of data storage efficiency. In order to have accurate positions in the reciprocal space, the histogram bins must be small. This means that the partially processed pre-histogrammed data could be much larger than needed to describe the measured raw counts. This is especially significant for inelastic scattering, where intensities are weak and most of the histogram bins may contain zeros. A typical 1 h measurement on ARCS, with 500 time or energy bins for each detector, has less than 2% nonzero bins. Keeping a sparse representation would, however, defeat the purpose of this approach, since consistent averaging requires all bins to be accounted for, even if they contain no counts (Peterson *et al.*, 2015 ▸).

### 3.2. Weighted-measurement approach to histogramming TOF data

As noted in previous papers (Michels-Clark *et al.*, 2016 ▸, 2017 ▸), the statistically correct way to combine data measured by multiple detectors and for different sample orientations is to replace equation (10) with

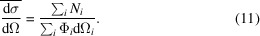

Here, 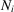 is the neutron count measured by the *i*th detector, with solid angle 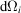 , and 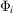 is the time-integrated neutron current through the sample during the measurement, *i.e.* the number of neutrons that hit the sample, which for a spallation neutron source is proportional to the total proton charge on the target. Although sample geometry can be important for determining 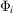 , here we assume homogeneous sample illumination by the neutron beam, deferring sample corrections to a separate publication. Similarly, for the inelastic scattering, in place of equation (9) we have

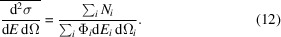




**Figure 4:**
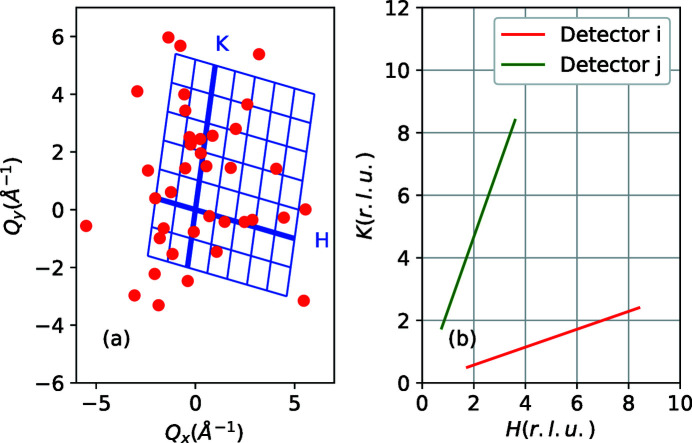
Geometry considerations in the weighted-measurement approach, based on equations (8) and (14). (*a*) The coordinates of neutron detection events (red dots) are stored in reciprocal coordinate space of the goniometer. A grid in 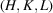 coordinates is obtained through affine transformations by rotating/scaling/skewing the axes (blue grid in the figure – the thick lines correspond to axes along the 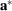 and 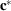 crystallographic axes). (*b*) In the reciprocal space, trajectories for each different detector or sample orientation (green and red curves correspond to some random detector positions or sample orientations) are straight lines, given by equation (14). The intersections with the reciprocal-lattice grid are obtained from equation (15). The statistical weight of the scattering from a particular detector, in each voxel, depends on the momentum (wavevector) associated with the intersection of the detector’s trajectory with the voxel’s boundary. Neither of the trajectories in the figure contribute to voxels with 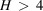 and 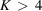 . Some voxels, such as the one closest to the origin, contain partial contributions from multiple detectors.

From the perspective of an end user who wants to analyze the histogrammed data, one needs to obtain the measured scattering cross section on a user-defined grid in reciprocal space. As noted in Section 1, this task can be split into two parts. The first step is to perform the coordinate transformation from the laboratory frame to the sample coordinate frame. This includes calculating the position where detection occurred, as well as other frame-dependent characteristics of each neutron detection event transformed from (detector position, time) instrument coordinates to (wavevector, energy) coordinates in the reciprocal space of the sample. As we discuss below, crystal symmetry operations, energy- and 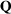 -dependent corrections related to the neutron beam optics (*e.g* deflection by a polarizer), energy-dependent transmission/absorption, temperature- and energy-dependent transformation to dynamic susceptibility *etc*. are all calculated in this first step, on the event level. The numerator in equations (11) and (12) is just a histogram representation of the counts, so each event is then assigned to the corresponding grid position [Fig. 4 ▸(*a*)].

Calculating the denominator is the second step. If the corresponding term for every configuration in the denominator is considered a weight, the number of counts in a bin from a particular configuration is just the cross section multiplied with the weight. Hence, the weights can be determined by measuring a standard sample with constant cross section in exactly the same configurations as the sample of interest. The number of counts from the standard sample provides a measurement of the corresponding weights.

### 3.3. An implementation of a weighted measurement

For a diffraction experiment, this approach was implemented by Michels-Clark *et al.* (2016 ▸) by measuring the incoherent elastic scatterer (vanadium) for each of the configurations in which the sample of interest was measured, following these steps:

(i) The TOF and detector position for the neutrons scattered from the sample were converted into sample reciprocal space.

(ii) The counts were then histogrammed onto a desired grid (‘data histogram’).

(iii) The above steps were repeated for a range of sample orientations, accumulating the counts in the data histogram.

(iv) A vanadium measurement was performed in the same configuration but for a single orientation; the number of counts in the vanadium data was adjusted for the ratio of the effective counting times (integrated proton charge of the accelerator was used for an integrated monitor count).

(v) The vanadium measurement was replicated in software by applying a set of the orientations of the goniometer used for the sample measurements.

(vi) The TOF and detector position for each vanadium neutron event were converted to the sample reciprocal space using the same UB matrix as for the sample to obtain the same orientation in reciprocal space.

(vii) Thus-transformed vanadium data were histogrammed on the same grid as the sample data, creating a ‘normalization histogram’.

(viii) The data histogram was divided by the normalization histogram, yielding a scattering cross section relative to that of vanadium.

**Figure 5:**
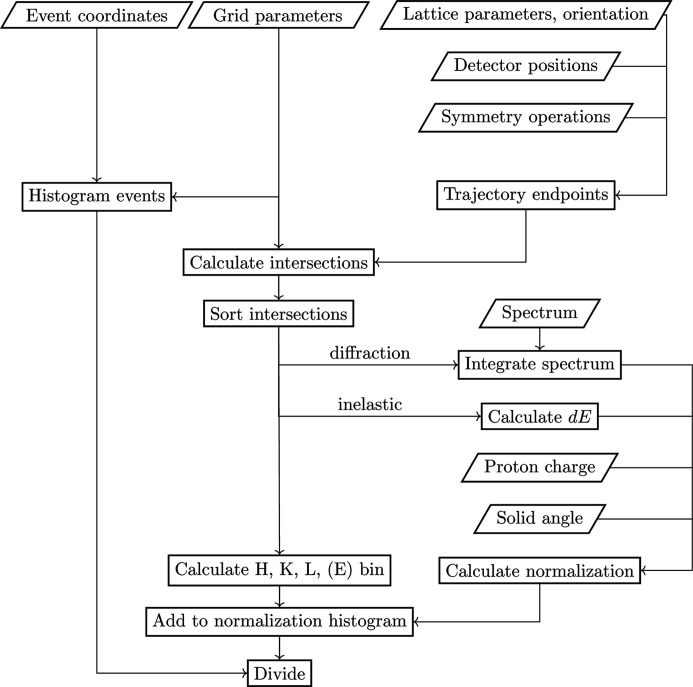
Algorithm workflow. The events (in reciprocal-space coordinates) are histogrammed to a regular grid. Detector positions, lattice parameters and sample orientations are used to calculate the trajectories in reciprocal space for each detector. The intersections of these trajectories with the given grid, together with counting time and solid-angle information, are used to calculate the statistical weights. Finally, the histogrammed event data are divided by the normalization (the weight) histogram, to obtain the normalized intensity histogram.

Results presented by Michels-Clark *et al.* (2016 ▸) are markedly less noisy and with smaller error bars than those obtained from the equal-weight treatment (see Figs. 4 and 5 in the cited work).

Although the weighted-measurement approach of Michels-Clark *et al.* (2016 ▸) is correct, that original implementation has three barriers for broader use. The first one is extensibility to inelastic scattering. While vanadium scatters uniformly in the momentum space, the scattering is elastic, *i.e.* dependence in the energy transfer direction is strongly peaked around 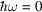 . The number of neutrons incoherently scattered by vanadium can no longer be used to measure the corresponding volume in the 4D sample reciprocal space in the denominator of equation (12). The second issue arises when the count rate from the incoherent scatterer is low for certain position(s) in reciprocal space. In this approach, each detector pixel carries its own information about the incident flux. Hence, for a finite measurement time of the standard sample, only a limited number of neutrons are available to measure the denominator terms in equation (11). One can, in principle, get better statistics by summing incoherent counts from detectors with the same energy-dependent response, but this is a computationally very expensive procedure requiring an efficient algorithm. This brings up the last issue with the original implementation, the amount of computing resources required to perform the calculations. Usually, the incoherent scatterer is measured for a sufficiently long time in order to have reasonable statistics. Replicating this measurement for every sample setting in order to perform the binning on the desired grid requires very large amounts of memory and processing power, which limits its applicability for some instruments.

## 4. Efficient weighted normalization and histogramming of the event TOF data: *MDNorm* algorithm

Here, we describe a faster, more memory-efficient way to calculate the normalization factors in the denominators of equations (11) and (12).

### 4.1. The normalization algorithm

In principle, the detector solid angle (measured experimentally or determined from geometrical considerations) is a property of each detected neutron event and the subsequent data processing does not in fact require histogramming. However, algorithms that completely avoid histogramming are a matter of future development. The algorithms developed at present, including the algorithm that we describe here, operate with creation, manipulation and analysis of the histogrammed data. In this case, the calculation of the weight factors for each voxel of a grid in the sample phase space is straightforward.

Theoretically, the elemental solid angle for each detected neutron can be calculated, knowing the detector size and position. In practice, however, it is more convenient (and in fact more reliable) to measure the effective detector solid angles experimentally, using an elastic incoherent scatterer, such as vanadium. Such measurement is time consuming because it requires a substantial number of neutrons to be counted by each detector in order to obtain low error bars on the measured detector solid angles. However, the measurement only needs to be performed once for each configuration of the instrument’s detector bank, and for most spectrometers it can also use white beam, which greatly enhances the incident flux. Thus measured detector solid angles can then be used for histogram weight calculation in equations (11) and (12).

The second ingredient for calculating the statistical weight in equations (11) and (12) is the total incident neutron current, 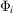 , used to measure each scattered neutron. For direct-geometry spectrometers at a spallation source, 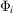 is just a number proportional to the total proton current supplied to the target during the measurement (at a given sample setting). For indirect-geometry spectroscopy and for TOF diffraction [equation (11)], the incident beam is not monochromatic and 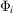 needs to be evaluated by the normalization algorithm. Due to the conservation of energy and momentum, neutrons with certain incident velocities and a given final energy are kinematically prohibited from scattering into some detectors. Hence, for a particular detector, *i*, the incident flux, 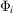 , must only include those incident neutrons that were kinematically allowed to scatter into the reciprocal-space region of interest containing that detector. In the case of diffraction, and partly for indirect-geometry spectroscopy, determining 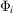 is the main task in calculating the statistical weight.

Finally, in the case of inelastic scattering, both direct and indirect, one needs to calculate the 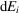 intervals for the neutron detection events. For a neutron detected within a given 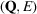 voxel of the histogram, 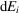 is the size of the energy transfer range that is constrained to the voxel by conservation of energy and momentum [equations (4) and (5)]. This is the length of the component along *E* for the detector trajectory [transformed from (detector position, time) to 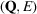 ], inside the voxel where the neutron was detected.

The new method described in this paper uses the fact that neutrons measured in a given detector, for a particular sample orientation, are located on a detector trajectory that is a straight line in the reciprocal space of the single-crystal sample. This can be seen from equation (8), which we rewrite as

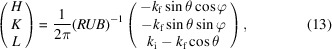




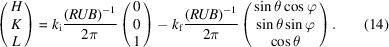

For a given orientation of the sample, *R*, and given angular coordinates of the detector, ( 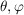 ), a vector of neutron (detector) coordinates in the reciprocal space of the crystal, 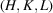 , is expressed as a linear combination of 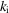 and 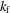 multiplied by some constant 3D vectors. In the case of single-crystal elastic scattering, we can write 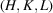 as a constant vector scaled by the incident neutron wavevector, 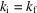 . Similarly, for inelastic spectroscopy, where either 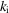 or 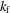 is fixed, 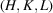 is a constant vector multiplied by the changing 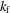 or 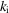 , plus another constant vector. A schematic representation of these trajectories is shown in Fig. 4 ▸(*b*).

We now consider measurement of the differential scattering cross section for a region of reciprocal space (voxel of a histogram) which is a cuboid with faces parallel to the crystallographic directions. We consider the changing wavevector being in the range bounded by the minimum, 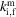 , and the maximum, 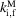 . Using these values in equation (14), we obtain the corresponding boundaries in the sample reciprocal space, 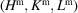 and 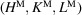 . We can then write the line equation for the neutron (detector) trajectory as

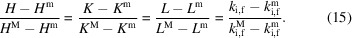




The coordinates of the intersection of a linear detector trajectory described by equation (15) and a plane at a constant value of *H*, *K*, *L*, or 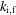 , are obtained by simply plugging that constant value into the equation. To calculate the differential scattering cross section, the important quantities to find are the values of 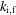 at the intersection points. For a cuboid reciprocal-space voxel, any detector trajectory that is not along a face or an edge can intersect the voxel at zero, one or two points. If we restrict the detector trajectory to be between 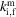 and 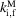 , the following cases have to be considered:

(i) Detector trajectory does not intersect the voxel – this means no contribution.

(ii) Detector trajectory intersects the voxel at exactly two points – we need to calculate the contribution of the line segment between the intersections.

(iii) Detector trajectory is completely inside the voxel – the contribution is the entire range between 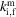 and 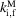 .

(iv) Detector trajectory intersects the voxel at exactly one point – this means that only one of 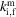 , 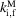 is inside the voxel and we need to calculate the contribution of a line segment between it and the intersection point.

If a trajectory is completely along a face or an edge of the voxel, it falls within one of the last three cases above. For example, if the trajectory is along a plane where the *L* component is a constant 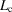 , then 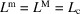 . Any intersection with the *H* or *K* planes will have the *L* component equal to 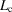 . The term corresponding to calculating the intersections along *L* will be of the form 0/0, does not provide any additional information and can be ignored.

In practical implementation, the algorithm works as shown in Fig. 5 ▸. First, the grid parameters of the histogram are read. Then, the neutron events are histogrammed to counts on this grid. Subsequently, in order to calculate the normalization in each voxel of the grid, the algorithm loops over all sample orientations, *R*, and over all detector positions, (θ, φ), and, for each detector trajectory and each sample orientation, computes the intersections with all the bounding planes of the grid, sorting them according to the 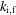 values. If there are none, it skips to the next detector. If there are intersections, then for the white-beam diffraction measurements the incident flux is integrated between the adjacent incident momentum values. For inelastic spectroscopy, the energy transfer range ( 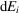 ) between the two bounding momentum values determined by the intersections is calculated using equation (5). Subsequently, for each detector, multiplication with the solid angle, 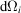 , is performed to obtain 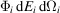 or 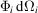 . These values are then accumulated on a grid congruent to the binned data. The final step is to divide the two arrays and thus obtain a histogram of the scattering cross section (up to an overall normalization).

### 4.2. Transformations of sample reciprocal space and symmetry operations on events

Clear delineation of the two steps in the workflow used in our approach, that of (i) operations on events and (ii) the eventual histogramming to a pre-defined grid of interest, provides significant advantages for data reduction and processing.

Firstly, the directions of interest in the sample reciprocal space, which define the grid for the histogrammed experimental data to be used for analysis, are not necessarily aligned with the crystallographic axes. Fortunately, the transformation from any three non-coplanar directions to the crystallographic ones is encoded in a simple 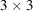 matrix, *W*, which can be applied to the event data. Specifically, if we substitute *RUBW* for *RUB* in our previous equations (13) and (14), then *H*, *K* and *L* are the coordinates along the new directions. It is important to emphasize that, by carrying out transformation on the event level instead of pre-histogrammed data, we avoid problems with non-trivial overlap of the histogram bins and the need to split the binned intensity among different voxels. We also avoid artifacts associated with the boundaries of the phase space covered by the measurement.

Similarly, it is often advantageous to apply crystal symmetry operations to the data. Any such operation in the reciprocal space can be written as a multiplication with a 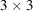 matrix applied after the *RUBW* product. By applying the symmetry operations, one can translate the measured neutron events to physically equivalent positions of the crystal’s reciprocal space. Using symmetry, one can markedly improve the statistics of the histogrammed data by replicating the measured events via application of the symmetry operations, *e.g.*


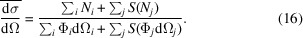

Here, *S* is the symmetry operator. At the histogramming stage, one counts all the neutrons detected in a reciprocal-space voxel, either directly or through symmetrization. The normalization algorithm then calculates the combined weight for the events measured by detectors in the direct measurement and detectors in symmetry-replicated positions, adds these weights together, and finally divides the total number of neutron counts by the total normalization factor.

From the implementation point of view, calculating the normalization for both non-axis-aligned grids and symmetry operations is done by simply calculating 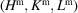 and 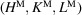 using the appropriate transformation matrix. The algorithm implementation in *Mantid* version 4.0.0 (Arnold *et al.*, 2014 ▸) takes full advantage of the space- and point-group description of symmetry operations.

## 5. Practical implementation and examples

### 5.1. Fast algorithm for flux integration

**Figure 6:**
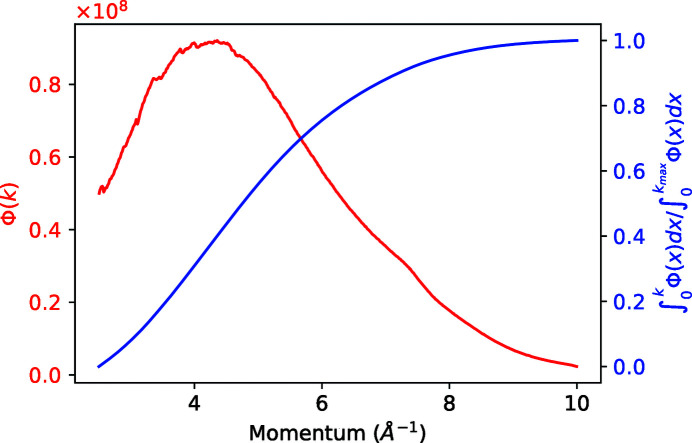
In order to quickly integrate the incident-momentum-dependent flux (red curve), a cached running integral is calculated using equation (17) (blue curve). Integrating the flux between two values is replaced by a lookup/ interpolation of the corresponding points on the running-integral curve and a simple subtraction.

As noted in the previous sections, on a white-beam instrument the statistical uncertainty of the incoherent scattering data used to measure the energy-dependent incident flux weighted by a detector solid angle, 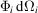 , might be less than satisfactory. In such cases, statistics for 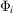 could be improved by adding together data from all detectors with the same energy response. For instruments like the CORELLI diffractometer at the SNS, the detectors are ^3^He tubes, with identical pressure and electronic data acquisition systems. By comparing the momentum-dependent spectra measured on vanadium, we found that all detector pixels can be summed together. On other instruments, such as the TOPAZ single-crystal diffractometer at the same facility, pixelated scintillator detectors are used whose energy response differs significantly from one detector bank to the next. Still, we can sum together all the events from a 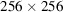 pixel area (TOPAZ has 25 such detectors, 15 × 15 cm in size). An example of integrated spectra is shown by the red curve in Fig. 6 ▸.

For every voxel of a histogram, the normalization algorithm needs to integrate the incident flux obtained from the vanadium measurement for all detector trajectories that intersect the voxel, between the corresponding momentum limits, 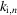 and 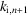 . Since the number of integrations is relatively large (10^6^–10^9^), we use a faster, interpolation method. We pre-compute the definite integral with a running upper bound, 

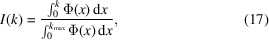

and store the result on a regular grid of about 1000 points, as shown by the blue curve in Fig. 6 ▸. Then, instead of performing a computationally expensive integration from 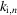 to 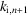 , we use linear interpolation of 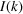 to obtain the values at the two intersection points. The integrated flux is given by 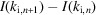 . The approximation is valid as long as the original flux dependence on 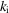 is relatively smooth (the variation of the flux between adjacent grid points is small compared with the flux).

### 5.2. Implementation notes

The algorithm is implemented as part of the *Mantid* (Arnold *et al.*, 2014 ▸) software package, under the name *MDNorm* (since version 4.0.0). The algorithm is implemented in C++, and it has a default Python binding and graphical user interface. The flux integration is done by the *MDNormSCDPreprocessIncoherent* algorithm. The event data, including all transformations performed in the first step of reduction, are stored in a multi-dimensional event (MDE) workspace, which can be histogrammed onto a grid of choice in 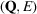 space. For each histogram (cut, slice, volume), the binned data are stored in a separate multi-dimensional histogram (MDH) workspace. The normalization and the calculated differential cross sections are also stored as MDH data sets. Examples using publicly available data can be found on the help page of the algorithm. Further developments will aim to integrate this algorithm in standard data processing workflows and graphical user interfaces.

### 5.3. Storage and usage of the neutron events

Originally, two versions of the workflow were implemented in C++, one for single-crystal diffraction and one for direct-geometry spectroscopy. To streamline keeping track of the start and end points of the trajectories, data were stored in *HKL* coordinates, which substantially complicated calculations for symmetry-related trajectories. Additionally, if the sample was misaligned, the original data coordinates had to be recalculated. So the decision was eventually made to store the data in an instrument 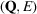 coordinate system tied to the goniometer ( 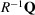 ).

While the format and structure of the MDE data are evolving, an important conceptual decision was to store detector information for each detected neutron event (sample orientation), even though most of the time this information is unchanged. Keeping the event information presents significant advantages for the data reduction, at a modest expense in additional memory. For example, it allows the sorting of events by detector property combined with other properties of the events – sample orientation, sample environment status *etc*. In contrast with earlier software packages, such as *Horace* (Ewings *et al.*, 2016 ▸) and *DAVE MSlice* (Azuah *et al.*, 2009 ▸), this allows one to reduce data sets with detectors masked only for a limited number of events (sample orientations), or even with moving detectors.

**Figure 7:**
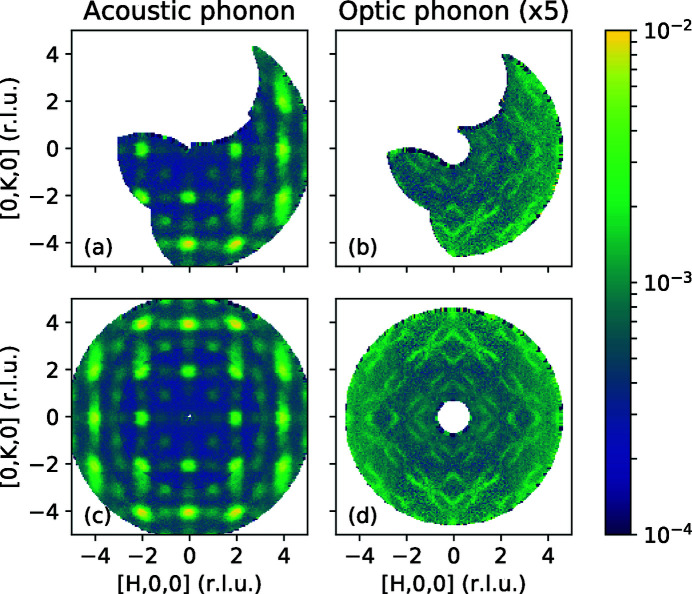
Measurement of the acoustic (*a*), 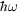 = 5 meV, and optic (*b*), 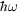 = 24 meV, phonons in FeTe_0.55_Se_0.45_. The HYSPEC instrument at SNS was used with two different detector positions. Panels (*c*) and (*d*) show the same data as in panels (*a*) and (*b*), respectively, symmetrized using the fourfold symmetry of the tetragonal crystal lattice.

The latter feature is essential for the HYSPEC instrument at SNS, which has position-sensitive detectors in a tank that rotates around the sample position in order to increase the reciprocal-space coverage. In Fig. 7 ▸, we show measurements on an iron-based superconductor, FeTe_0.55_Se_0.45_ (Fobes *et al.*, 2016 ▸). To cover a large reciprocal-space volume, we used two different detector tank positions (32 and 92°). To accommodate the offset, data were collected for slightly different sample rotation angle ranges. The constant energy slices at 5 and 24 meV show acoustic and optic phonons [panels (*a*) and (*b*), respectively]. In both slices one can see a seamless transition between data measured at different detector angles (a slightly higher background for high-angle detector position is an intrinsic feature of the data, not the reduction). Panels (*c*) and (*d*) show the same data symmetrized using the fourfold symmetry of the crystal structure in the 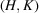 plane of the presented slices.

**Figure 8:**
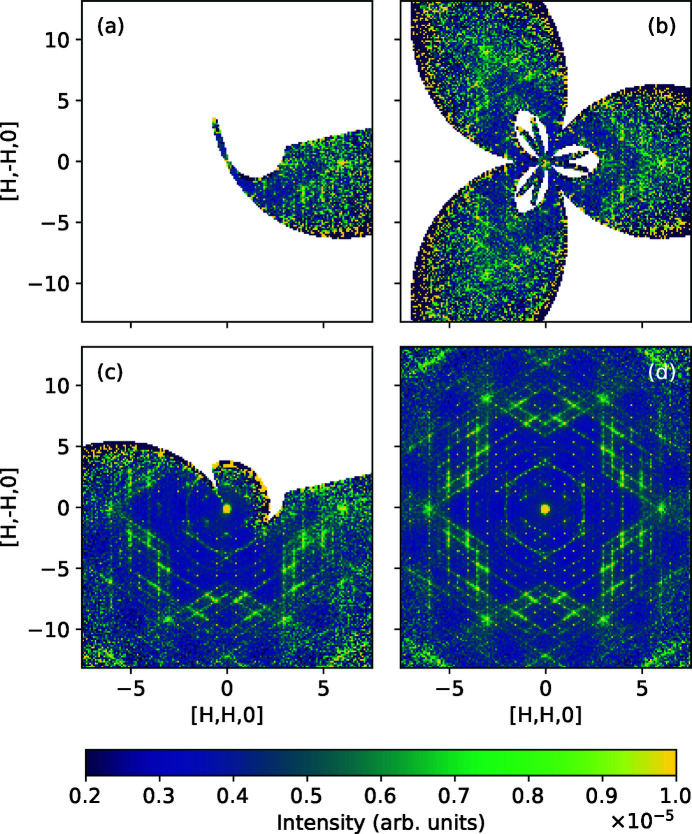
Diffuse scattering from benzil at 100 K collected with CORELLI at SNS. (*a*) Single goniometer position; (*b*) single goniometer position with symmetry applied; (*c*) multiple (rotated 180° in steps of 5°, 36 total) different goniometer positions combined; (*d*) multiple different goniometer positions combined and symmetry applied.

Among other examples highlighting the advantages of the *MDNorm* algorithm is the measurement of diffuse scattering from benzil at CORELLI at the SNS (Welberry & Whitfield, 2018 ▸). In order to observe diffuse scattering, which for benzil can be 10^3^–10^4^ times weaker than Bragg scattering, using symmetry and the correct normalization are essential. Benzil belongs to space group 152 ( 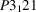 ), which has six different site symmetries: 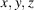 ; 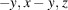 ; 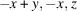 ; 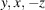 ; 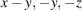 ; 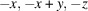 (Arnold *et al.*, 2016 ▸). The data were collected at 100 K for 36 different goniometer positions, by rotating the sample 180° around the vertical axis in 5° steps. As shown in Fig. 8 ▸, applying symmetry allows a more complete reciprocal-space coverage and improves statistics.

### 5.4. Intensity comparison of data reduction methods for inelastic scattering

The statistically correct normalization and seamless symmetrization of the histogrammed intensity are especially important for the polarized neutron TOF data, such as those obtained at the HYSPEC instrument at the SNS (Winn *et al.*, 2015 ▸; Zaliznyak *et al.*, 2017 ▸). Firstly, the scattering intensities are low. This is not only because each polarized cross section is only a fraction of the unpolarized one but primarily because there is an intensity penalty of one to two orders of magnitude associated with the polarized beam neutron optics. Secondly, things are complicated by corrections associated with the polarized beam optics, such as the scattered beam deflection by the supermirror transmission polarization analyzer and its energy- and angle-dependent transmission. Finally, the scattering intensity in each polarization channel [spin-flip (SF) and non-spin-flip (NSF)] has to be corrected for the finite polarization efficiency encoded in the flipping ratio (FR). Namely, the ‘net’ SF/NSF intensity is given by the difference in the measured ‘raw’ intensities, appropriately weighted using the FR (Zaliznyak *et al.*, 2017 ▸). In making such intensity difference histograms, it is absolutely essential that statistical errors of the measured data are correctly propagated.

**Figure 9:**
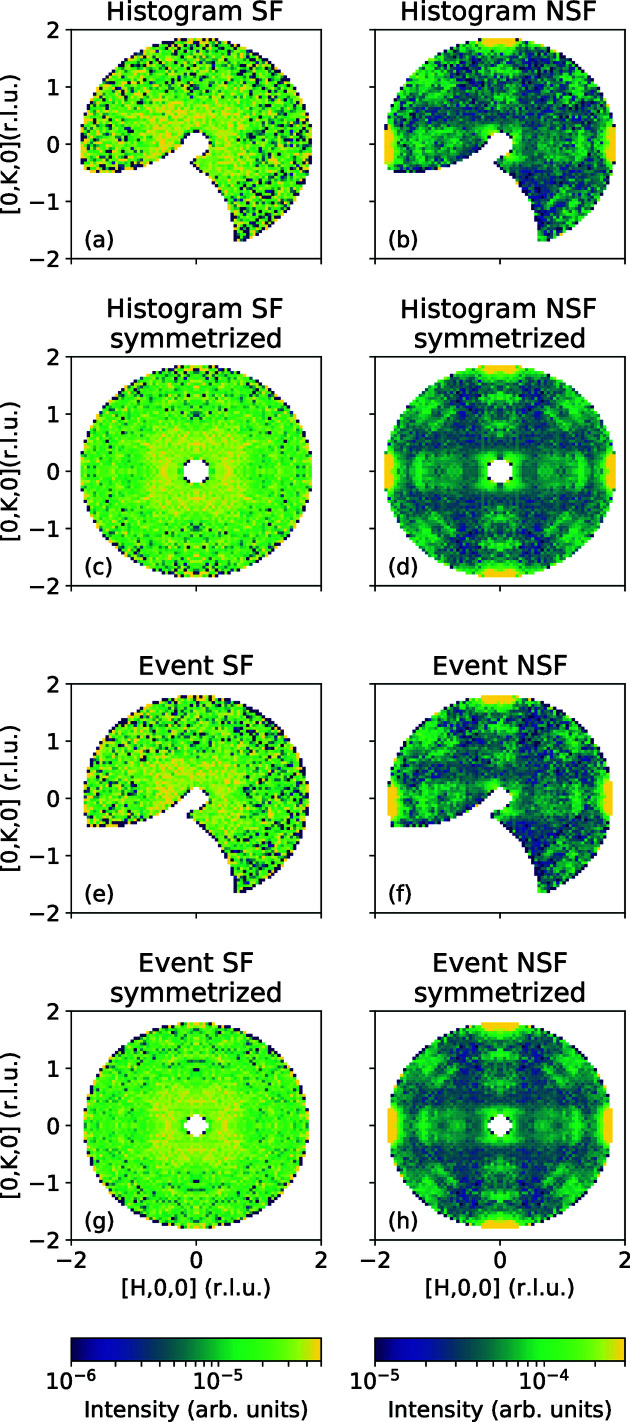
Comparison of equal-statistics measurements of polarized scattering in FeTe_0.55_Se_0.45_ at 575 K. Spin-flip (SF) data are shown on the left, non-spin-flip (NSF) on the right. Data shown in panels (*a*)–(*d*) were processed using a pre-histogramming in energy transfer, while the new approach was used for panels (*e*)–(*h*). The 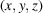 , 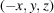 , 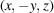 and 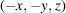 symmetry operations were applied in panels (*c*), (*d*), (*g*) and (*h*). Since each orientation was measured with the same integral flux, there is very little difference between pre-histogramed and event-based methods.

In the case where the energy bins are small, the detector efficiencies are comparable and the incident flux for each orientation is the same, there should be very little difference between intensities obtained from the pre-histogramming and event-based approaches. Such is the case in Fig. 9 ▸. We show the polarized measurement on the iron-based superconductor, FeTe_0.55_Se_0.45_, studied by Li *et al.* (2021 ▸). Panels (*a*) and (*b*) show the SF and the NSF intensity slice at 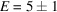 meV reduced using the *Mslice* workflow. A similar reduction using our new approach is presented in panels (*e*) and (*f*), correspondingly. Panels (*c*) and (*d*) show the same data symmetrized in histogram mode, while panels (*g*) and (*h*) show the same intensity symmetrized using our *MDNorm* algorithm. Still, careful inspection of the symmetrized data does reveal a somewhat better performance of the event approach.

**Figure 10:**
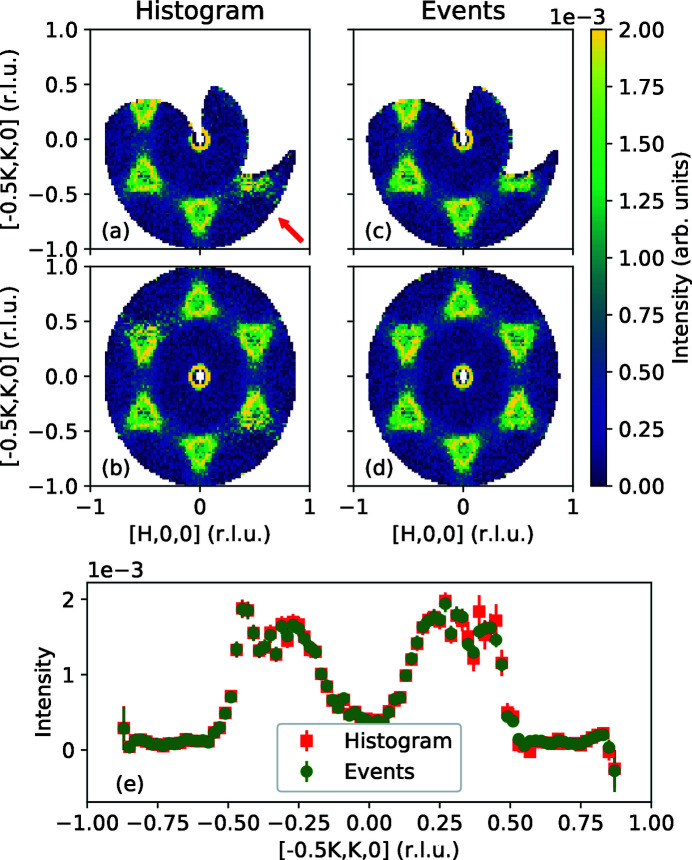
Comparison of unequal-statistics measurements in KYbSe_2_. Here, 180 orientations, 1° apart, were measured for 400 s each. In addition, the first 30 s in the first 30 orientations (above the red arrow) were processed separately and added to the data. (*a*) Pre-histogramming approach, not symmetrized. (*b*) Pre-histogramming approach, symmetrized (inversion). (*c*) Event approach, not symmetrized. (*d*) Event approach, symmetrized (inversion). (*e*) Comparison of cuts on symmetrized data for the two approaches at 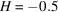 , showing larger error bars for the pre-histogrammed approach in regions where low-statistics data are added to higher-statistics ones.

In cases where the statistics of the measurements are widely different, the fluctuations in intensities in the overlap regions are expected to be dominated by the measurements with low flux, similar to the results in the diffraction case (Michels-Clark *et al.*, 2016 ▸). To emphasize this point, we show inelastic scattering in the KYbSe_2_ triangular antiferromagnet (Scheie *et al.*, 2021 ▸). In the original experiment, 180 orientations, 1° apart, were measured for 400 s each. In addition, for the first 30 orientations we processed data separately for the first 30 s each, to simulate shorter runs. Fig. 10 ▸ shows how the addition of the simulated lower time measurements affects the intensity pattern. In the pre-histogrammed approach, the lower-right corner in panel (*a*) is noisier than the corresponding region for the event-based approach [panel (*c*)]. In addition, the symmetrization (in this case a simple inversion) for the pre-histogrammed approach is negatively affecting the quality of the original data in the upper-left corner [panel (*b*)], compared with the similar region in the event-based data treatment [panel (*d*)]. To get a better feel for the noise associated with the addition of a lower-statistics measurement, we show a comparison of a cut on symmetrized data for the two approaches, at 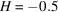 [panel (*e*)]. The error bars for the pre-histogrammed approach are larger than those for the event-based approach in the region around 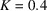 .

### 5.5. Background subtraction

In some cases, the background (BG) depends on the angular position of the sample and is measured in a rotation scan, similarly to the signal. In such cases, it is straightforward to subtract the two histograms produced from the corresponding event data sets that were obtained for similar rotation ranges. In many cases, however, the BG is, to a very good approximation, independent of the crystal orientation and is measured for a single sample rotation angle. This is the case for scattering from most sample environments, or for ambient BG neutrons. One approach to subtract such a BG, measured at a single angle from a signal measured in a sample rotation scan, is to replicate the BG measurement for all sample orientations. This approach was implemented in the early versions of our MDE data reduction workflow. It is similar to that used by Michels-Clark *et al.* (2016 ▸) described in Section 3.2. We found that this process is slow and often very memory intensive. When replicated to 120 sample orientations, a 2 h BG measurement is equivalent to a 10 day neutron data collection and contains a correspondingly large number of events. While this procedure still works for instruments with smaller detector banks, such as HYSPEC, it is prohibitively memory intensive for spectrometers with large detector arrays, such as ARCS, CNCS or SEQUOIA at the SNS.

We devised an extension to the algorithm which avoids replication of the single-angle BG data; this feature was introduced in a recent update (*Mantid 6.1.0*). In the new version, the histogramming of the BG measurement is done for each individual sample orientation, using the fact that the statistical weight for both the sample and the BG data is the same, up to a simple scaling by the ratio of incident neutron count. If for each sample setting the data are measured for the same amount of time as the BG, the new approach takes about 20% longer than in the case of no BG, compared with a 100% overhead using the previous procedure. The 20% figure is an empirical estimate of the time required to re-histogram the BG events onto the same grid as the data. The remaining 80% is a measure of the calculations for statistical weights, which is not required in this new approach.

**Figure 11:**
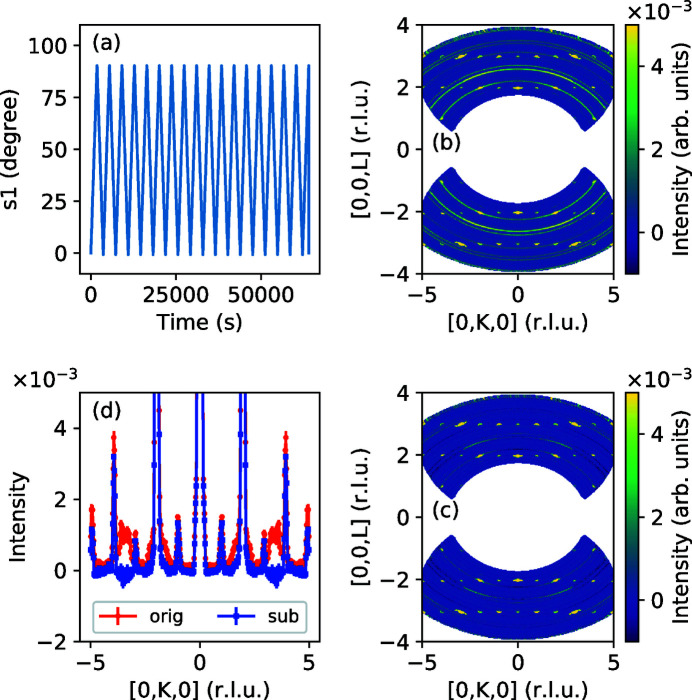
(*a*) Event-based data acquisition allows for filtering detected neutrons, in this case on the goniometer rotation angle around the vertical axis (s1). (*b*) Elastic scattering in the horizontal plane. Data are symmetrized using reflections about the 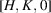 and 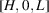 planes. (*c*) A 0.5°-width region around s1 = 49° is used as background. The efficient subtraction method used is described in the text. (*d*) Detail cut at 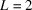 shows the removal of the sample environment background (aluminium powder lines).

As another example, we consider removing the time-independent BG from a continuous rotation measurement. The BG consists of ambient neutrons or electronic noise that contributes a constant count rate, independent of the TOF, measurement time or sample orientation. Data in Fig. 11 ▸ were measured on the HYSPEC spectrometer using the continuous rotation method, and the scattering events were filtered by sample orientation [the sample angle, S1, is shown in panel (*a*)]. Usually, the time-independent BG is calculated at each sample rotation from a portion of the energy spectrum where sample scattering is kinematically prohibited and contains only a small number of scattering events. In our case, due to the limited measurement time for each orientation, the number of BG neutrons is small but still comparable to the number of counts in the inelastic spectrum. This can lead to a noisy BG subtraction. Instead, here we used all the rotations to create a data set containing only time-independent scattering, which was therefore measured for a long period of time, including all the sample orientations. The relative counting error for the BG is now an order of magnitude less than that for the data, allowing for a smooth low-noise subtraction.

The application of this approach is even more important for single-angle or synthetic BG measurements, such as carried out by Leiner *et al.* (2019 ▸), where the BG measurement time is more than one order of magnitude longer than the data collection time at each sample setting.

### 5.6. Further performance optimization – remarks about computational approaches

Two changes to the initial implementation improved parallel scaling and run time. Performing the loop over detectors in parallel may create simultaneous writes to elements in the signal array. Initially, writes were synchronized with a critical section allowing only one write to any element of the array at a time. Thread contention was reduced by moving synchronization to individual elements in the signal array. This was done by making the signal array a std::vector<std::atomic<double>> with updates being performed using std::atomic<double>::compare_exchange_weak. This could be simplified in C++20 with the additional specializations for floating-point types.

The *MDNorm* algorithm creates temporary normalization and data workspaces for each measurement that are then added to the final result. Allocating and de-allocating memory on the heap are expensive and unnecessary operations. Additional inputs to pass optional temporary data and normalization workspaces were added to *MDNorm*. Small changes to users’ scripts eliminate these costly operations. If unspecified, blank workspaces initialized to 0 are created and the behavior is unchanged. Existing scripts continue operating as expected.

## 6. Conclusion

In conclusion, we developed an efficient and statistically correct approach for reducing and histogramming diffuse elastic and inelastic TOF neutron scattering data. The governing principle of this approach is that the data are only histogrammed once and thus all deficiencies and hurdles of re-histogramming are avoided. The reduction workflow consists of two distinct parts. (i) Operations on the properties of the measured neutron events, such as their position in the reciprocal space of a single-crystal sample, the position on the detector array, the duration of the measurement *etc*, which are carried out prior to histogramming. These include, but are not limited to, coordinate transformations, symmetrization, sorting, masking, positional and intensity corrections, and other operations that are independent of the grid to which the data are eventually binned. (ii) The normalization algorithm, which uses the linear property of detector trajectories in the reciprocal space of a single-crystal sample. This algorithm performs statistically correct weighting of all the events located in a particular voxel of reciprocal space. The algorithm creates a histogrammed representation of the measured neutron scattering cross section with correctly propagated statistical errors of the measured intensities.

The approach described in this paper is implemented in the algorithm *MDNorm* and a helper algorithm *MDNormSCDPreprocessIncoherent* as part of the *Mantid* (Arnold *et al.*, 2014 ▸) software package and its usage for processing the SNS data is gaining momentum. A future version of the algorithm will implement the same calculations for measurements on indirect-geometry spectrometers. Extending the approach to inelastic scattering from powders is possible but, surprisingly, less straightforward, because the trajectories are no longer linear in the reciprocal space.

## References

[bb1] Arnold, H., Aroyo, M. I., Bertaut, E. F., Burzlaff, H., Chapuis, G., Fischer, W., Flack, H. D., Glazer, A. M., Grimmer, H., Gruber, B., Hahn, T., Klapper, H., Koch, E., Konstantinov, P., Kopsky, V., Litvin, D. B., Looijenga-Vos, A., Momma, K., Muller, U., Shmueli, U., Souvignier, B., Spence, J. C. H., de Wolff, P. M., Wondratschek, H. & Zimmermann, H. (2016). *International Tables for Crystallography*, Vol. A, *Space-Group Symmetry*, edited by M. I. Aroyo, pp. 193–687. Chichester: Wiley.

[bb2] Arnold, O., Bilheux, J. C., Borreguero, J. M., Buts, A., Campbell, S. I., Chapon, L., Doucet, M., Draper, N., Ferraz Leal, R., Gigg, M. A., Lynch, V. E., Markvardsen, A., Mikkelson, D. J., Mikkelson, R. L., Miller, R., Palmen, K., Parker, P., Passos, G., Perring, T. G., Peterson, P. F., Ren, S., Reuter, M. A., Savici, A. T., Taylor, J. W., Taylor, R. J., Tolchenov, R., Zhou, W. & Zikovsky, J. (2014). *Nucl. Instrum. Methods Phys. Res. A*, **764**, 156–166.

[bb3] Azuah, R. T., Kneller, L. R., Qiu, Y., Tregenna-Piggott, P. L. W., Brown, C. M., Copley, J. R. D. & Dimeo, R. M. (2009). *J. Res. Natl Inst. Stand. Technol.* **114**, 341. 10.6028/jres.114.025 PMC4646530 27504233

[bb4] Berry, K. (2016). *The Neutron Lifecycle Lecture Series*, Lecture 6a. SNS, ORNL, Oak Ridge, USA. https://conference.sns.gov/event/56.

[bb5] Bewley, R., Eccleston, R., McEwen, K., Hayden, S., Dove, M., Bennington, S., Treadgold, J. & Coleman, R. (2006). *Physica B*, **385–386**, 1029–1031.

[bb6] Busing, W. R. & Levy, H. A. (1967). *Acta Cryst.* **22**, 457–464.

[bb40] Coldea, R. (2000). *MSlice: Matlab Visualisation Software for Single Crystal and Powder Time-of-Flight Neutron Data*, https://www.isis.stfc.ac.uk/Pages/mslice-manual.pdf.

[bb7] Ewings, R., Buts, A., Le, M., van Duijn, J., Bustinduy, I. & Perring, T. (2016). *Nucl. Instrum. Methods Phys. Res. A*, **834**, 132–142.

[bb8] Ewings, R. A., Stewart, J. R., Perring, T. G., Bewley, R. I., Le, M. D., Raspino, D., Pooley, D. E., Škoro, G., Waller, S. P., Zacek, D., Smith, C. A. & Riehl-Shaw, R. C. (2019). *Rev. Sci. Instrum.* **90**, 035110. 10.1063/1.5086255 30927771

[bb9] Fobes, D. M., Zaliznyak, I. A., Tranquada, J. M., Xu, Z., Gu, G., He, X.-G., Ku, W., Zhao, Y., Matsuda, M., Garlea, V. O. & Winn, B. (2016). *Phys. Rev. B*, **94**, 121103.

[bb10] Giacovazzo, C. E. (1992). *Fundamentals of Crystallography.* IUCr/Oxford University Press.

[bb11] Granroth, G. E., An, K., Smith, H. L., Whitfield, P., Neuefeind, J. C., Lee, J., Zhou, W., Sedov, V. N., Peterson, P. F., Parizzi, A., Skorpenske, H., Hartman, S. M., Huq, A. & Abernathy, D. L. (2018). *J. Appl. Cryst.* **51**, 616–629.

[bb12] Inamura, Y., Nakatani, T., Suzuki, J. & Otomo, T. (2013). *J. Phys. Soc. Jpn*, ** 82** (Suppl. A), SA031.

[bb13] Jogl, G., Wang, X., Mason, S. A., Kovalevsky, A., Mustyakimov, M., Fisher, Z., Hoffman, C., Kratky, C. & Langan, P. (2011). *Acta Cryst.* D**67**, 584–591. 10.1107/S090744491101496X PMC3107055 21636899

[bb14] Kajimoto, R., Nakamura, M., Inamura, Y., Mizuno, F., Nakajima, K., Ohira-Kawamura, S., Yokoo, T., Nakatani, T., Maruyama, R., Soyama, K., Shibata, K., Suzuya, K., Sato, S., Aizawa, K., Arai, M., Wakimoto, S., Ishikado, M., Shamoto, S.-i., Fujita, M., Hiraka, H., Ohoyama, K., Yamada, K. & Lee, C.-H. (2011). *J. Phys. Soc. Jpn*, ** 80** (Suppl. B), SB025.

[bb15] Keen, D. A., Gutmann, M. J. & Wilson, C. C. (2006). *J. Appl. Cryst.* **39**, 714–722.

[bb16] Leiner, J. C., Jeschke, H. O., Valentí, R., Zhang, S., Savici, A. T., Lin, J. Y. Y., Stone, M. B., Lumsden, M. D., Hong, J., Delaire, O., Bao, W. & Broholm, C. L. (2019). *Phys. Rev. X*, **9**, 011035.

[bb17] Li, Y., Zaki, N., Garlea, V. O., Savici, A. T., Fobes, D., Xu, Z., Camino, F., Petrovic, C., Gu, G., Johnson, P. D., Tranquada, J. M. & Zaliznyak, I. A. (2021). *Nat. Mater.* **20**, 1221–1227. 10.1038/s41563-021-00984-7 33888904

[bb18] Lumsden, M. D., Robertson, J. L. & Yethiraj, M. (2005). *J. Appl. Cryst.* **38**, 405–411.

[bb19] Michels-Clark, T. M., Savici, A. T., Lynch, V. E., Wang, X., Chodkiewicz, M., Weber, T., Bürgi, H.-B. & Hoffmann, C. M. (2017). *J. Appl. Cryst.* **50**, 1559. 10.1107/S1600576717006781 PMC5627685 29021738

[bb20] Michels-Clark, T. M., Savici, A. T., Lynch, V. E., Wang, X. P. & Hoffmann, C. M. (2016). *J. Appl. Cryst.* **49**, 497–506. 10.1107/S1600576716001369 PMC4815874 27047306

[bb21] Nakajima, K., Ohira-Kawamura, S., Kikuchi, T., Nakamura, M., Kajimoto, R., Inamura, Y., Takahashi, N., Aizawa, K., Suzuya, K., Shibata, K., Nakatani, T., Soyama, K., Maruyama, R., Tanaka, H., Kambara, W., Iwahashi, T., Itoh, Y., Osakabe, T., Wakimoto, S., Kakurai, K., Maekawa, F., Harada, M., Oikawa, K. E., Lechner, R., Mezei, F. & Arai, M. (2011). *J. Phys. Soc. Jpn*, ** 80** (Suppl. B), SB028.

[bb22] Perring, T. (1999). Personal communication (unpublished).

[bb23] Perring, T., Taylor, A., Osborn, R., Paul, D., Boothroyd, A. & Aeppli, G. (1994). *Proceedings of ICANS XII*, pp. 1–60. RAL Report 94-025. Rutherford Appleton Laboratory, Didcot, UK. http://neutronresearch.com/parch/1993/01/199301010600.pdf.

[bb24] Peterson, P. F., Campbell, S. I., Reuter, M. A., Taylor, R. J. & Zikovsky, J. (2015). *Nucl. Instrum. Methods Phys. Res. A*, **803**, 24–28.

[bb25] Peterson, P. F., Olds, D., Savici, A. T. & Zhou, W. (2018). *Rev. Sci. Instrum.* **89**, 093001. 10.1063/1.5034782 30278744

[bb26] Scheie, A. O., Ghioldi, E. A., Xing, J., Paddison, J. A. M., Sherman, N. E., Dupont, M., Sanjeewa, L. D., Lee, S., Woods, A. J., Abernathy, D., Pajerowski, D. M., Williams, T. J., Zhang, S.-S., Manuel, L. O., Trumper, A. E., Pemmaraju, C. D., Sefat, A. S., Parker, D. S., Devereaux, T. P., Movshovich, R., Moore, J. E., Batista, C. D. & Tennant, D. A. (2021). *arXiv*:2109.11527.

[bb27] Shirane, G., Shapiro, S. M. & Tranquada, J. M. (2002). *Neutron Scattering with a Triple-Axis Spectrometer: Basic Techniques.* Cambridge University Press.

[bb28] Squires, G. L. (2012). *Introduction to the Theory of Thermal Neutron Scattering*, 3rd ed. Cambridge University Press.

[bb29] Stone, M. B., Niedziela, J. L., Abernathy, D. L., DeBeer-Schmitt, L., Ehlers, G., Garlea, O., Granroth, G. E., Graves-Brook, M., Kolesnikov, A. I., Podlesnyak, A. & Winn, B. (2014). *Rev. Sci. Instrum.* **85**, 045113. 10.1063/1.4870050 24784665

[bb30] Van Hove, L. (1954). *Phys. Rev.* **95**, 249–262.

[bb31] Welberry, R. & Whitfield, R. (2018). *QuBS*, **2**, 2.

[bb32] Welberry, T. R., Gutmann, M. J., Woo, H., Goossens, D. J., Xu, G., Stock, C., Chen, W. & Ye, Z.-G. (2005). *J. Appl. Cryst.* **38**, 639–647.

[bb33] Windsor, C. (1981). *Pulsed Neutron Scattering.* London: Taylor & Francis.

[bb34] Winn, B., Filges, U., Garlea, V. O., Graves-Brook, M., Hagen, M., Jiang, C., Kenzelmann, M., Passell, L., Shapiro, S. M., Tong, X. & Zaliznyak, I. (2015). *EPJ Web Conf.* **83**, 03017.

[bb35] Ye, F., Liu, Y., Whitfield, R., Osborn, R. & Rosenkranz, S. (2018). *J. Appl. Cryst.* **51**, 315–322. 10.1107/S160057671800403X PMC5884386 29657565

[bb36] Zaliznyak, I. A. & Lee, S.-H. (2005). *Modern Techniques for Characterizing Magnetic Materials*, edited by Y. Zhu, pp. 3–64. Boston: Springer US.

[bb37] Zaliznyak, I. A., Savici, A. T., Ovidiu Garlea, V., Winn, B., Filges, U., Schneeloch, J., Tranquada, J. M., Gu, G., Wang, A. & Petrovic, C. (2017). *J. Phys. Conf. Ser.* **862**, 012030.

